# Hypothermic machine perfusion before viability testing of previously discarded human livers

**DOI:** 10.1038/s41467-021-21182-8

**Published:** 2021-02-12

**Authors:** Otto B. van Leeuwen, Yvonne de Vries, Vincent E. de Meijer, Robert J. Porte

**Affiliations:** Department of Surgery, Section of Hepatobiliary Surgery & Liver Transplantation, University Medical Center Groningen, University of Groningen, Groningen, Netherlands

**Keywords:** Bile ducts, Phase I trials, Translational research

**Arising from** H. Mergental et al. *Nature Communications* 10.1038/s41467-020-16251-3 (2020)

The results from the VITTAL study, with back-to-base end-ischemic normothermic machine perfusion (NMP) for viability testing of high-risk donor livers, were eagerly awaited as in this field different techniques are currently being used^1^. Of 31 initially discarded livers, 22 (71%) met the viability criteria during NMP and were transplanted with a 100% 90-day graft survival; however, three out of ten livers donated after circulatory death (DCD) developed biliary complications (30%) and required retransplantation. Because the trial was already initiated in 2016, more recent insights in viability testing should be considered when discussing the design and results of this clinical trial. Therefore, there are three issues that needs commenting to interpret the high rate of biliary complications reported: (1) the viability criteria used in this trial, (2) production of bile during NMP and (3) the use of end-ischemic NMP alone.

## Cholangiocyte viability assessment and liver function biomarkers for clinical decision making during NMP

In contrast to hypothermic (<12 °C) machine perfusion (HMP), livers are metabolically fully active during NMP. This leads to restoration of hepatobiliary metabolism, such as clearance of lactate, production of (coagulation) proteins, and, importantly, bile production. Two types of viability criteria for donor livers during NMP have been distinguished: hepatocellular (‘liver parenchyma’) and cholangiocellular (‘bile duct’) viability criteria (Table 1). In the current study by Mergental et al., only hepatocellular viability criteria were used to assess ‘transplantability’ of liver grafts during NMP^1^. The first reports on the value of bile composition as a tool to determine bile duct viability were published after the initiation of this study^2,3^. In our experience, a substantial number of especially high-risk DCD grafts do not meet the cholangiocellular viability criteria during NMP (Table 1). We therefore do not transplant these livers, as bile composition during NMP has been shown to correlate with biliary injury and thereby the risk of post-transplant cholangiopathy^4^. Transplanting high-risk (DCD) donor livers without monitoring bile composition during NMP poses a risk to the recipient, as other investigators have previously shown a high rate of post-transplant cholangiopathy after end-ischemic NMP of high-risk DCD livers^3,5^. Therefore, with the current knowledge it is not a complete surprise that Mergental et al. have noted a high incidence of post-transplant cholangiopathy (30% after DCD transplantation) and need for retransplantation in their series^1^.

**Table 1:** Hepatocellular and cholangiocellular viability criteria for human donor livers during NMP^a^.

VITTAL criteria	Groningen criteria	
Hepatocellular criteria	Hepatocellular criteria	Cholangiocellular criteria
Lactate clearance	Lactate clearance	Delta bile pH
*At least two of the following criteria*	Perfusate pH stabilization	Delta bile bicarbonate
	Bile production	Delta bile glucose
- Bile production		
- Perfusate pH stabilization		
- Metabolism of glucose		
- Stable flows		
- Homogenous perfusion		

^a^The VITTAL criteria had to be met within 4 h of NMP, the Groningen criteria within 2.5 h of NMP^1,8^.

In another clinical trial on NMP and transplantation of initially discarded human donor livers (DHOPE-COR-NMP trial), we have monitored bile composition *point of care*^6–8^. Following an early case of post-transplant cholangiopathy, we have learned that absolute values of bile pH, bicarbonate, and glucose during NMP are not the most suitable markers for cholangiocellular viability testing. We have learned that the difference between the bile and perfusate levels of pH, bicarbonate, and glucose should be used to identify alkalization of the bile and reabsorption of glucose by the biliary epithelium (biomarkers of bile duct viability; Table 1)^8^. Currently, we have performed over 50 DHOPE-COR-NMP procedures of initially nation-wide discarded, high-risk DCD livers and after using the delta pH, bicarbonate, and glucose as cholangiocellular viability criteria during NMP, we have not observed any case of post-transplant cholangiopathy.

## Bile production during NMP

In the study by Mergental et al., 23% of the livers did not appear to produce bile^1^. In our experience, we have always been able to explain low or absent bile flow during NMP by a technical issue with the biliary drain. Regularly, the tip of the biliary drain can be stuck against the bile duct wall, leading to obstruction of the lumen and limiting bile flow. We have tested biliary drains extensively in preclinical experiments, and observed that bile flow initiates more rapidly when a small diameter drain is used, as a result of increased capillary force. As a result, we have been using a small feeding tube catheter (8 French) with an open tip and side holes as a biliary drain^9^.

## Combination of end-ischemic HMP and NMP versus NMP alone

In the study by Mergental et al., it was chosen to perform end-ischemic NMP only^1^. Since the VITTAL study was initiated in 2016, other centers shared similar experiences showing high percentages of biliary complications after NMP for high-risk (DCD) donor livers^3,5^. End-ischemic NMP exposes a donor organ to ischemia-reperfusion injury, to which the bile ducts are extremely sensible. It has been shown before that a short period of dual hypothermic oxygenated machine perfusion (DHOPE) reduces reperfusion injury of the liver and biliary tree^10^. Several studies have demonstrated a beneficial effect of performing DHOPE prior to either direct transplantation or NMP^11–15^. After combined DHOPE and NMP, hepatic adenosine triphosphate content and biliary bicarbonate and bilirubin were higher in comparison to livers that underwent NMP only^15^. As a result, in 2017, the DHOPE-COR-NMP trial was initiated with a combination of one-hour DHOPE, followed by one-hour controlled oxygenated rewarming, and subsequent NMP, with the aim to reduce ischemia-reperfusion injury at the start of NMP^6–8^. Interestingly, all livers in the DHOPE-COR-NMP trial met the hepatocellular viability criteria used in the VITTAL study, despite the fact that all livers were derived from high-risk DCD donors with a substantially higher median age, compared to the VITTAL study^8^. This could suggest that a short period of DHOPE prior to NMP is very effective in preventing additional reperfusion injury of the liver parenchyma (hepatocytes) at the start of NMP. Only livers that have suffered too much (warm) ischemic injury of the bile ducts during and after the DCD procedure may no longer be salvageable with DHOPE and will not meet the biliary viability criteria during the NMP phase.

In conclusion, in a rapidly advancing field of liver machine perfusion, we are all still learning and it is through sharing of our experiences that we can collective determine the most optimal use of the new technology in the benefit or our patients. From the emerging evidence, it becomes clear that end-ischemic NMP is an important tool to assess and select donor livers that, based on traditional criteria, were initially considered not suitable for transplantation. Yet, end-ischemic NMP alone, as applied in the VITTAL study, does not protect the bile ducts against ischemia-reperfusion injury. Therefore, a short period of HMP prior the NMP, as well as the use of cholangiocellular viability criteria during machine perfusion, are important modifications that may help avoid post-transplant morbidity and graft loss due to cholangiopathy.
